# Limits to the evolution of dispersal kernels under rapid fragmentation

**DOI:** 10.1098/rsif.2021.0696

**Published:** 2022-03-23

**Authors:** Gili Greenbaum, Efrat Dener, Itamar Giladi

**Affiliations:** ^1^ Department of Evolution, Ecology, and Behavior, The Hebrew University of Jerusalem, Jerusalem 9190401, Israel; ^2^ Albert Katz International School for Desert Studies, Jacob Blaustein Institutes for Desert Research, Ben Gurion University of the Negev, Sede Boqer Campus, Midreshet Ben-Gurion, Israel; ^3^ Mitrani Department of Desert Ecology, Swiss Institute for Dryland Environmental and Energy Research, Jacob Blaustein Institutes for Desert Research, Ben-Gurion University of the Negev, Midreshet Ben-Gurion 8499000, Israel

**Keywords:** dispersal evolution, dynamic fragmentation, quantitative traits, evolutionary constraints, ecological modelling

## Abstract

Adaptive evolution of dispersal strategies is one mechanism by which species can respond to rapid environmental changes. However, under rapid anthropogenic fragmentation, the evolution of dispersal may be limited, and species may be unable to adequately adapt to fragmented landscapes. Here, we develop a spatially explicit model to investigate the evolution of dispersal kernels under various combinations of fragmentation dynamics and initial conditions. We also study the consequences of modelling an evolutionary process in which dispersal phenotypes continuously and gradually shift in phenotype space in a manner corresponding to a polygenic underlying genetic architecture. With rapid fragmentation rates, we observed the emergence of long-term transient states in which dispersal strategies are not well suited to fragmented landscapes. We also show that the extent and length of these transient states depend on the pre-fragmentation dispersal strategy of the species, as well as on the rate of the fragmentation process leading to the fragmented landscape. In an increasingly fragmented world, understanding the ability of populations to adapt, and the effects that rapid fragmentation has on the evolution of dispersal, is critical for an informed assessment of species viability in the Anthropocene.

## 1. Introduction

Human-induced habitat loss and fragmentation are currently affecting many species across most terrestrial ecosystems [1–5]. Under anthropogenic fragmentation, the loss of habitat is rapid and often culminates in a patchy, fragmented landscape [3,6]. The ability of species to evolve in the face of rapid fragmentation, and in particular the ability to evolve dispersal strategies adaptive to highly fragmented landscapes, is crucial for their survival in the Anthropocene [4,5]. The evolution of dispersal strategies may be affected by several factors, such as the spatial characteristics of the landscape [7,8], the genetic architecture underlying dispersal traits [9] and the characteristics of the fragmentation process. Models of eco-evolutionary dynamics that explore how different factors affect the evolution of dispersal under fragmentation are, therefore, essential for understanding the impact and consequences of fragmentation [4,10]. Importantly, incorporating the way in which dispersal phenotypes change based on the underlying genetic architecture of dispersal traits, as well as the dynamics of the fragmentation process, is crucial for understanding the evolution of dispersal under rapid fragmentation rates.

Classic modelling of dispersal evolution in patchy landscapes focused on the evolution of dispersal rate of sessile organisms, without accounting for dispersal distances [11–16]. Another family of models specifically tracks the evolution of dispersal kernels, which are described either by a parametric probability function [7,17–19], or as a non-parametric distribution of probabilities for dispersal to a set of discrete distance classes [9,20–24]. In general, many models demonstrate that in fragmented landscapes, unless there is temporal heterogeneity in habitat suitability, a short-distance dispersal strategy is likely to evolve [18,25], although the evolution of polymorphic dispersal strategies and of fat-tailed dispersal kernels can also emerge [7,20,26,27]. Most models focus on the emergence of an evolutionarily stable dispersal strategy in an already fragmented landscape. Implicitly, this means that fragmentation is being considered as a state rather than being modelled as a process, that dispersal strategies evolve in a static landscape [27], and that the evolutionary trajectories leading to stable states are of little importance because equilibrium conditions are reached on relatively short timescales.

Although static-landscape models are informative regarding which dispersal phenotype is expected to eventually evolve in fragmented landscapes, the extent to which species are able to adaptively evolve such a fragmentation-adapted dispersal kernel when the fragmentation process is rapid is yet unclear (by ‘fragmentation-adapted’, we mean the phenotype which would have evolved in the fragmented landscape given sufficient time and given no constraints on the evolutionary exploration of the phenotype space). In other words, even after the fragmentation process has ended, long-term transient states may emerge. In addition, the evolutionary process may be sensitive to, and potentially limited by, the dispersal strategies that different species already have at the onset of the fragmentation process. Such evolutionary constraints can be reinforced by the underlying genetic architecture of the dispersal phenotype. In polygenic dispersal phenotypes determined by many small-effect loci, the evolutionary exploration of the dispersal phenotype space is likely to be gradual and continuous. By contrast, when only a few large-effect loci are involved, mutations may allow a more non-continuous exploration of phenotype space. Therefore, due to constraints in the exploration of the phenotypic space, under rapid fragmentation, transient dispersal kernels that are not fragmentation-adapted may remain for a substantial period.

Previous models have assumed mutation models in which progeny may have dispersal phenotypes which, compared to their parents, allow for a substantial shift in the shape of their dispersal kernel [20,22,28]. These model designs were intentional, with the aim of allowing the most advantageous dispersal strategies to evolve unconstrained (most models, for the purpose of tractability, avoid modelling plasticity by assuming identity between genotypes and phenotypes; we follow suit). However, these models implicitly assume that large-effect mutations are possible and common, and are therefore more appropriate for genetic architectures with large-effect loci. Nevertheless, many dispersal phenotypes are related to quantitative traits such as seed weight, size, shape or length [9], and therefore evolving dispersal kernels are expected to shift continuously and gradually through the phenotype space of dispersal strategies, rather than to spontaneously evolve new types of substantially different dispersal strategies.

The implementation of continuous-shifting exploration of the phenotype space (i.e. where mutations induce continuous quantitative changes between phenotypes, as opposed to mutations allowing ‘jumps’ between considerably different phenotypes) in models of dispersal evolution is important, as it reflects the presumably polygenic and composite nature of many dispersal phenotypes, which emerge from the interaction of several non-discrete traits [9]. Consequently, it introduces biologically relevant limitations to the evolution of dispersal. This continuous treatment of phenotype exploration has two important implications for modelling the evolution of dispersal. First, depending on the initial pre-fragmentation dispersal phenotypes, the evolution of dispersal phenotypes that are adaptive in fragmented landscapes may take more time to evolve with a continuous-shifting phenotype exploration, because evolutionary trajectories need to traverse more intermediate phenotypes in order to ‘discover’ the adaptive phenotype. Second, intermediate dispersal phenotypes between the initial phenotypes and the adaptive phenotypes may be maladaptive under different conditions [28–30], and thus generate additional barriers in phenotype space at various stages of the fragmentation process. Such barriers could further slow down the adaptive evolution of dispersal, extending the transient states of dispersal phenotypes in the fragmented landscape. The extent to which populations are able to adapt to a fragmented landscape, therefore, is expected to depend on the similarity between the initial pre-fragmentation phenotype and the fragmentation-adapted phenotype in the fragmented landscape, as well as on the rate and characteristics of the fragmentation process itself.

Here, we explore the evolution of dispersal kernels of sessile organisms under dynamic fragmentation, and we consider how the evolution of dispersal is affected by the genetic architecture underlying dispersal (continuous-shifting phenotypes versus unconstrained mutations), by the initial pre-fragmentation dispersal strategy, and by the rate of fragmentation. We conduct simulations in which the landscape and the fragmentation process are explicitly modelled, based on a previously described simulation framework [20] to allow for comparison with previous results. However, we model fragmentation as a process, rather than a state, and we incorporate a continuous-shifting phenotype model, with gradual shifts in the shape of the dispersal kernels, which captures the quantitative-trait nature of many dispersal phenotypes. The aim of our investigation is to examine whether limitations on evolution emerge and induce long-term transient states in which dispersal strategies are not adapted to the fragmented landscape. Understanding these limits is important to understand the potential for adaptive evolution to occur in response to the rapid fragmentation of the Anthropocene within reasonable timescales.

## 2. Methods

**Figure 1.**
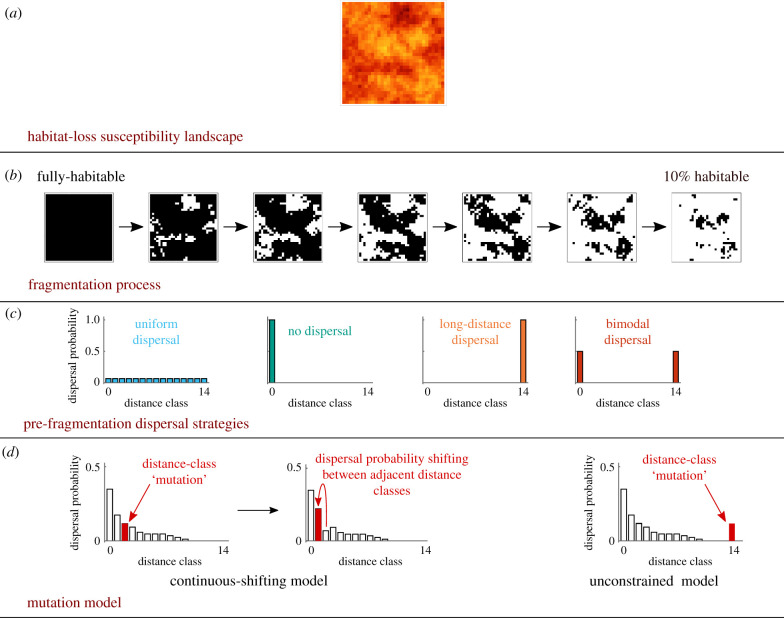
Schematic description of our model. (*a*) An autocorrelated torus landscape is generated, with values in each cell in the landscape representing the susceptibility for loss of habitability of that cell. (*b*) A fragmentation process is generated based on habitat-loss susceptibility, from a fully inhabitable landscape to a landscape with only 10% inhabitable cells. Cells with high loss susceptibility values become uninhabitable earlier than those with lower values. (*c*) Each simulation is initiated with a homogeneous population with the same pre-fragmentation dispersal strategy. We investigate evolutionary dynamics starting from four types of pre-fragmentation strategies. (*d*) In the continuous-shifting mutation model, each ‘mutation’ event is constrained such that the probability of dispersal shifts to an adjacent distance class. In the unconstrained model, mutations in different dispersal classes are independent.

We explore the evolution of dispersal strategies under fragmentation using an individual-based evolutionary simulation model (figure 1). For simplicity and tractability, we consider a population of a non-sexually reproducing organism with passive dispersal, as a typical representative of sessile organisms. We also assume the absence of phenotypic plasticity. Initially, the population occupies a continuously inhabitable landscape, represented by a two-dimensional array of cells which individuals may occupy. Some of the cells will become uninhabitable as fragmentation proceeds (figure 1*b*). At the pre-fragmentation state, all individuals have an identical dispersal strategy for their offspring (figure 1*c*). We simulate a fragmentation process in which some parts of the landscape become uninhabitable (figure 1*b*). During this process, we simulate a birth–death process, local extinction and colonization that results in the replacement of individuals, and a mutation model that governs the evolution of the dispersal kernels (figure 1*d*). We track this evolutionary process at the population level, by averaging dispersal phenotypes over all the individual dispersal kernels, at each point in time. All simulations were coded in Mathematica v. 12.3.

### 2.1. Birth–death process

The birth–death process is simulated in a similar manner to that in Hovestadt *et al*. [20]. Each inhabitable cell accommodates a single individual at each point in time. The lifespan of each individual was drawn from a normal distribution with mean = 100 time-steps and *σ* = 5. In other words, time is scaled such that each time-step is one-hundredth of the life expectancy of an individual. We therefore use ‘life expectancy’ as a biologically interpretable time unit for our model. The initial age distribution in each simulation is heterogeneous, by assigning each individual an age that is randomly drawn from a uniform distribution on the interval from 1 to 125. When an individual dies, it is replaced by a seed randomly selected from all seeds arriving at the vacated cell, following the dispersal dynamics described below.

### 2.2. Generating landscapes and fragmentation process

We generated neutral autocorrelated landscapes on a 32 × 32 torus using a method based on spectral synthesis [31]. Initially, before fragmentation occurs, all cells are inhabitable, and each cell is assigned a numeric value indicating the cell's habitat-loss susceptibility (figure 1*a*). The assignment of these values is not spatially random, but is governed by the landscape patchiness, which is characterized by a Hurst index [20,31]. Spatial autocorrelation was set to 0.2 in all simulations, which generates modest amounts of autocorrelation (alternative parameterizations in electronic supplementary material).

We then simulate a fragmentation process in which cells that were assigned higher habitat-loss susceptibility values become uninhabitable first, and as fragmentation progresses more cells become uninhabitable, at a constant rate (figure 1*b*). The fragmentation process is halted when only 10% of the landscape is inhabitable, representing the final fragmented landscape (alternative parametrizations in electronic supplementary material). We considered three fragmentation rates: slow, which occurs over 90 000 time-steps (900 times the typical life expectancy, henceforth ‘life expectancy time units', from fully habitable landscape to 10% inhabitable landscape, or 0.001% loss per time-step); intermediate, over 9000 time-steps (90 ‘life expectancy time units’); and rapid, over 900 time-steps (9 ‘life expectancy time units’). In this way, we explore fragmentation rates at three different orders of magnitude.

### 2.3. Dispersal kernels

In our model, each individual is characterized by a dispersal kernel, which describes the relative portions of its seeds that disperse to each distance class. In order to allow dispersal kernels to evolve unconstrained by an *a priori* parametric shape function, we model dispersal kernels as discrete distributions of dispersal distance classes, following Hovestadt *et al*. [20]. For each distance class, *i*, *d_i_* represents the proportion of seeds that are dispersed to distance *i* cell lengths, with *d*_0_ representing the portion of non-dispersed seeds; we denote the full dispersal kernel as the vector ***d***. Therefore, each cell in the landscape at distance *i* from an individual receives a portion *e_i_* = *d_i_*/*g_i_* of the seeds from that individual, where *g_i_* is the number of cells at distance *i* from a focal cell (electronic supplementary material, figure S3A; see electronic supplementary material for treatment of cells at non-integer distances). We simulated dispersal kernels with 15 distance classes ($d=(d_{0},\ldots ,d_{14})$; $\sum_{i=0}^{14}d_{i}=1$). The maximal dispersal distance, *i* = 14, allows for long-range dispersal across approximately half of the landscape area.

At the outset of each simulation, all individuals are initiated with the same dispersal kernel. In order to better understand the relevance of pre-fragmentation dispersal strategies for dispersal kernel evolution, we explore four types of initial dispersal kernels that denote extreme types of dispersal strategies (figure 1*c*): (i) non-dispersal, in which all seeds remain in the parent cell (*d*_0_ = 1, *d_i_* = 0 for *i* ≥ 1); (ii) uniform dispersal, in which all distance classes have the same probability (*d_i_* = 1/15 for all 0 ≤*i* ≤ 14; this is also the dispersal strategy that evolves in the initial, fixed, continuous landscape in our model; electronic supplementary material, figures S1 and S2); (iii) long-distance dispersal, in which all seeds disperse to the maximal distance (*d*_14_ = 1, *d_i_* = 0 for *i* ≤ 13); and (iv) bimodal dispersal, in which seed dispersal probabilities are equally split between the lowest (no dispersal) and the maximal distance class (*d*_0_ = 1/2, *d*_14_ = 1/2 and *d_i_* = 0 for 1 ≤ *i* ≤ 13). These dispersal types represent four dissimilar phenotypes, which do not necessarily reflect biologically realistic dispersal strategies, but rather were intended to represent extremely different initial starting points in the phenotype space.

### 2.4. Dispersal dynamics

To determine which seed replaces an individual that has died, the seed-arrival distribution for the vacated cell is computed based on the current dispersal kernels of all individuals in the landscape and their distances from the vacant cell. Thus, for a vacated cell, the seed-arrival distribution is *S* = (*s*_1_, …, *s_n_*), where *n* is the current number of individuals in the simulation, the seed contribution for individual *k* is $s_{k}=e_{i_{k}}$, where *i_k_* is the distance between individual *k* and the vacated cell (measured as Euclidean distance between centres of cells, rounded to nearest integer), and $e_{i_{k}}=d_{i_{k}}/g_{i}$ is the portion of seeds distributed to a cell at distance *i_k_* by individual *k* (*g_i_* is the number of cells at distance *i* from a focal cell; electronic supplementary material, figure S3A). We then pick one seed from the distribution *S* to inhabit the vacated cell. Notice that *S* also includes the offspring of the individual that has died in the vacated cell, so a non-dispersed offspring may replace its parent. By describing the seed-arrival distribution in this manner, we account for dispersal-related seed mortality, since in each distance class seeds may end up in either inhabitable or uninhabitable cells, depending on the landscape configuration at that point in time. This distance-related mortality, which emerges from the process, is the only dispersal-related cost in our model. Therefore, as the simulated landscapes are spatially autocorrelated, seeds dispersing to short distances are likely to remain within the parental patch and arrive at a suitable habitat, whereas long-distance dispersal of seeds comes at the cost of likely arriving to an unsuitable habitat, but also provides the opportunity for colonizing distant patches.

### 2.5. Continuous-shifting mutation model

Progeny dispersal phenotypes (i.e. their dispersal kernels) may be different from their parent's phenotypes due to genetic mutations. We model the changes in dispersal strategies as ‘mutations’, which are overall phenotypic changes between parents and offspring in dispersal probabilities. In our continuous-shifting mutation model, we account for the fact that dispersal kernels are typically a composite consequence of one or several quantitative traits (e.g. seed mass, seed size, flight-organ shape, etc.). Accordingly, we assumed that changes in dispersal phenotypes are gradual, and that the dispersal kernel can only shift dispersal probability between adjacent dispersal classes rather than mutations occurring independently in different distance classes (figure 1*d*). Consequently, the evolutionary process is constrained by the existing dispersal kernel. For example, if an individual has a maximal dispersal distance of 10 (*d_i_* = 0 for *i* > 10), then in the continuous-shifting model its offspring might have a maximal dispersal distance of 11 or 9, but not 14 or 4, which would require many mutations of very large-effect sizes to occur in a single reproduction event, unrealistic for a polygenic quantitative trait.

For a dispersal kernel ***d***, the offspring dispersal kernel ***d***′ is determined by the following procedure. (i) For each distance class *i*, we determine whether it ‘mutates’, at probability *μ*. (ii) If class *i* mutates, we randomly select adjacent distance class *i* ′ to be *i* − 1 or *i* + 1 (if *i ′* < 0 or *i* ′ > 14, no mutation occurs). (iii) We determine mutation magnitude − *m* < *m*′ < *m*, uniformly at random. (iv) We define $d_{i}^{c}=d_{i}+m^{\prime }$ and $d_{i^{\prime }}^{c}=d_{i^{\prime }}-m^{\prime }$ (if for any distance class $d_{i^{\prime }}^{c}<\;0$ or $d_{i}^{c}>\;1$, we set $d_{i^{\prime }}^{c}=0$ or $d_{i}^{c}=1$, respectively). In other words, we add *m*′ to *d_i_*, and subtract *m*′ from $d_{i^{\prime }}.\;$(v) To attain the final progeny dispersal kernel ***d***′ we normalize the dispersal kernel to sum to 1, i.e. $d_{i}^{\prime }=d_{i}^{c}/\sum_{i=0}^{14}d_{i}^{c}$ for all *i*.

Overall, this procedure shifts dispersal probability between adjacent distance classes, following the parameters *μ* (rate of shifting events) and *m* (magnitude of shift), as depicted in figure 1*d*. This mutation model allows for non-parametric flexibility, as specific distance classes may be selected for or against, yet it constrains evolution to follow gradual shifts in the shape of dispersal kernel phenotypes.

We set the mutation rate to be *µ* = 2/15, meaning that on average two distance classes change per reproduction event, and the maximal mutation effect size to be *m* = 0.5 (alternative parametrization in electronic supplementary material).

### 2.6. Unconstrained mutation model

To contrast the continuous-shifting mutation model and compare with previous studies (e.g. [20]), we simulated the same scenarios with a mutation model that is not constrained to shift dispersal probabilities between adjacent distance classes. In this model, each dispersal class can mutate independently of adjacent distance classes: (i) for each distance class *i*, we determine whether it mutates, at a probability *μ*; (ii) we determine mutation magnitude − *m* < *m*′ < *m*, uniformly at random; (iii) we define $d_{i}^{\prime }=d_{i}+m^{\prime }$ (if $d_{i}^{\prime }<\;0$ or $d_{i}^{\prime }>\;1$, we assume $d_{i}^{\prime }=0$ or $d_{i}^{\prime }=1$, respectively); and (iv) we normalize the dispersal kernel to sum to 1 to attain the progeny dispersal kernel ***d***′. In other words, the procedure is similar to the one in the continuous-shifting model, only that dispersal probabilities do not necessarily shift between adjacent distance classes, but rather the dispersal probability in each distance class can increase or decrease independently of probabilities in other distance classes, as depicted in figure 1*d*. We keep the mutation rate *μ* and the magnitude *m* at the same values as for the respective continuous-shifting model.

### 2.7. Simulation scheme

In each simulation, we generate a landscape, draw an initial age distribution and then simulate the fragmentation process, the birth–death and dispersal processes, and the mutation model, as described above (figure 1). For each combination of the four initial kernels and three fragmentation rates, we run 200 simulation replicates for 1100 ‘life expectancy time units' (110 000 time-steps). This time scale allows populations at least 200 ‘life expectancy time units’ in the fragmented state of 10% landscape habitability (900 ‘life expectancy time units' for the slow fragmentation process plus 200 additional time units). During the simulation, we record in each time-step the mean dispersal kernel, by averaging for each distance class *i* the dispersal proportions *d_i_* over all individuals living at that time. We also document the mean dispersal-related mortality at each time-step, defined as the proportion of the seeds that are dispersed to uninhabitable cells averaged across all individuals living at that time.

To coherently interpret the results, we also conducted several control simulations. To determine the effect of landscape fragmentation, we simulated the dynamics in (i) an identical manner as presented above, except that there is no fragmentation process (i.e. the landscape remains entirely inhabitable) and (ii) fragmentation is instantaneous, not gradual (electronic supplementary material). We also conduct corresponding simulations for all scenarios with the constrained mutation model, to evaluate the evolutionary impact of incorporating the continuous-shifting mutation model.

## 3. Results

**Figure 2.**
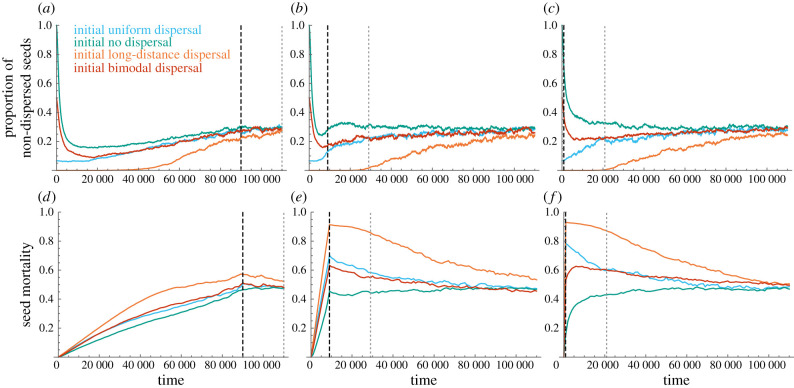
Evolutionary trajectories of mean non-dispersed seeds (*d*_0_) and mean seed mortality, with the continuous-shifting model. Mean *d*_0_ and mortality across the population are averaged over 200 simulation replicates. (*a*,*d*) Slow fragmentation; (*b*,*e*) Intermediate-rate fragmentation; (*c*,*f*) Rapid fragmentation. Different initial pre-fragmentation dispersal strategies are shown in different colours. The thick dashed black lines delineate the end of the fragmentation process (10% inhabitable cells), and the thin grey dotted lines delineate 200 life-expectancies in the fragmented landscape.

**Figure 3.**
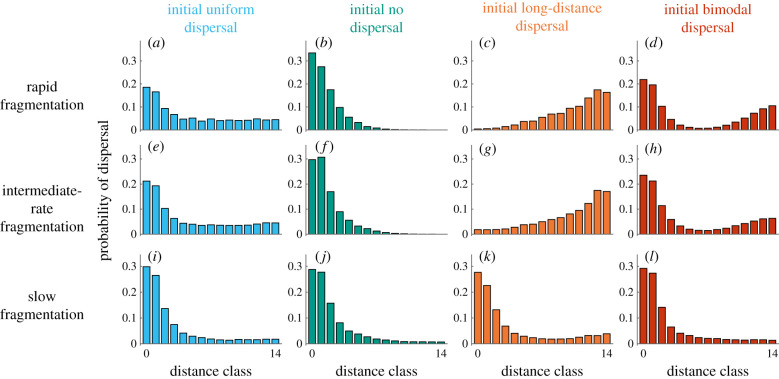
Evolved dispersal kernels following 200 life-expectancies in the fragmented state. The panels show the mean dispersal kernel across the population corresponding to the times denoted by dotted lines in figure 2, for each fragmentation rate and initial dispersal kernel. With rapid (*a*–*d*) and intermediate (*e*–*h*) fragmentation rates, the evolved dispersal kernels after 200 life-expectancies are different for different initial dispersal phenotypes and are relatively similar to the initial dispersal kernels. With slow fragmentation (*i*–*l*), the dispersal kernels evolve to a similar short-distance dispersal strategy irrespective of the initial pre-fragmentation dispersal kernel.

When modelling evolution as a continuous-shifting process in phenotype space, we observe long-term transient dispersal phenotypes that extend throughout and after the fragmentation process, which are dependent on initial pre-fragmentation phenotypes (figure 2). Considering a population going through a fragmentation process and then inhabiting a fragmented landscape of 10% habitability for 200 life-expectancies (dotted lines in figure 2), we observe that with rapid and intermediate fragmentation rates, the transient states result in dispersal phenotypes that are still relatively similar to the initial pre-fragmentation phenotypes (figure 3*a*–*h*). However, for slow fragmentation rates, 200 life-expectancies following the fragmentation process short-distance dispersal kernels evolve regardless of initial conditions (figure 3*i–l*), with less than 0.21 probability of dispersal to distance above 7 $(\sum_{i>7}d_{i}\leq 0.21$ for all initial conditions). The transient states persist for long periods after the fragmentation process has halted, and evolutionary trajectories eventually converge to a fragmentation-adapted population-averaged dispersal kernel in which the proportion of non-dispersers is approximately *d*_0_ = 0.3, and seed mortality rates are around 0.5 (figure 2). These mortality rates represent the trade-off between dispersal to non-habitable cells and the opportunities of colonizing other patches in the fragmented landscape through longer range dispersal, and are different for different landscape characteristics (electronic supplementary material, figures S10–S13). Variation among simulation replicates was mostly of similar magnitude for different initial conditions, although slightly larger with initially bimodal dispersal (electronic supplementary material, figure S16).

**Figure 4.**
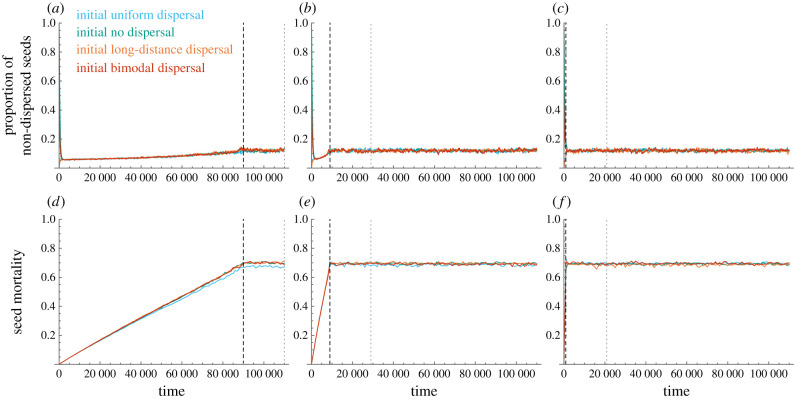
Evolutionary trajectories of non-dispersed seeds and seed mortality with the unconstrained model. The figure corresponds to figure 2, but with the unconstrained mutation model rather than the continuous-shifting mutation models. The thick dashed black lines delineate the end of the fragmentation process (10% inhabitable cells), and the thin grey dotted lines delineate 200 life-expectancies in the fragmented landscape.

The long-term transient states that were observed for rapid and intermediate fragmentation rates when modelling evolution of dispersal as a continuous shift in phenotype space (figures 2 and 3) were not observed when modelling evolution using the unconstrained mutation model (figure 4). With this evolutionary model, rapid convergence to a fragmentation-adapted phenotype is reached, irrespective of the fragmentation rate or the initial pre-fragmentation dispersal phenotypes (figure 4).

From the four initial dispersal phenotypes we simulated, the phenotype most resembling the evolved short-distance fragmentation-adapted dispersal phenotype is the initial no-dispersal phenotype (green in figures 1*c* and 3), and the most different is the long-distance dispersal (red in figures 1*c* and 3), in terms of the proportion of dispersal concentrated on short-distance classes. Accordingly, the dispersal kernels that evolved (measured 200 life-expectancies after cessation of the fragmentation process) were most similar to the fragmentation-adapted phenotype with initial no-dispersal, and least similar to initial long-range dispersal phenotypes (green and red in figure 3). The distance in phenotype space between the initial phenotype and the fragmentation-adapted phenotype also correlated with the extent and length of the temporal transient states, with initial long-range dispersal resulting in particularly divergent and long-lasting transient states (green and red curves in figure 2). With the two initial phenotypes with ‘intermediate’ distance in phenotype space to the fragmentation-adapted phenotype, uniform and bimodal dispersal kernels, the transient states are also intermediate in terms of their length and extent (blue and yellow in figures 2 and 3).

The dependency of the transient states on initial dispersal phenotypes and fragmentation rates remained with different parametrization of the model, but these states were longer and more divergent with low mutation rates than with high mutation rates (figures 2 and 3 compared to electronic supplementary material, figures S6–S9). They were also longer and more divergent with more landscape patchiness (figures 2 and 3 compared to electronic supplementary material, figures S10–S13) and were less pronounced when the final fragmented landscape was more habitable (figures 2 and 3 compared to electronic supplementary material, figures S14 and S15).

Control simulations of evolution in a fully habitable landscape demonstrated that the evolved averaged dispersal phenotypes across the population are fairly uniform (electronic supplementary material, figure S1), as expected with a population experiencing only random drift without selection (no mortality). However, biases for specific distance were observed, which we attribute to the discretization of space in our model (electronic supplementary material, figure S3). These biases, therefore, do not represent biologically meaningful biases, but rather are a result of discretization of space. The distance class specific biases are not observed in the simulations with fragmentation, and their effect seems secondary to actual selection processes on dispersal in our simulations. Control simulations with instantaneous fragmentation (electronic supplementary material, figures S4 and S5) generate results very similar to those seen with rapid fragmentation (figures 2*c*,*f* and 3*a–d*).

## 4. Discussion

The environmental crisis that accompanies human-induced climate change, habitat loss and habitat fragmentation introduces urgency to our attempts to monitor, predict and potentially mitigate the undesired changes to the distribution of species on Earth. As part of these efforts, we are experiencing a dramatic increase in research efforts aimed at documenting, modelling and improving our understanding of eco-evolutionary dynamics in fragmented landscapes, with the research of dispersal playing a pivotal role in those efforts [4,5,32–34]. It has long been recognized that the ability of species to persist in fragmented landscapes is tightly linked to life-history traits, especially those related to dispersal abilities [35,36]. Here, we expand on previous modelling efforts [20,22,37,38] by incorporating evolutionary dynamics suited to modelling dispersal phenotypes governed by polygenic genetic architectures, as well as a dynamic fragmentation of the landscape. Our model is, obviously, just an abstraction of the complexity of the natural system, but it adds to our knowledge by highlighting the implications of some simplifying assumptions that are commonly made by previous models. Specifically, our results demonstrate that (i) the ability for timely adaptation to fragmentation depends on the characteristics of the fragmented landscape, as well as on historical pre-fragmentation characteristics of the population and the dynamics of the deterioration of the landscape, and (ii) modelling evolution as a process where shapes of dispersal kernels are restricted to shift continuously in phenotype space is important when studying evolutionary responses to fragmentation in species where dispersal phenotypes have underlying polygenic architecture.

The temporal extent of the transient states we observe, as well as the divergence of evolutionary trajectories in these states, depends on the initial conditions of the population. The transient states last longer for initial dispersal kernels with more weight in longer distances than for other initial dispersal kernels. This is because, in a highly fragmented landscape, a high probability for short-distance dispersal is important to avoid high-seed mortality (electronic supplementary material, figure S17), and because the transient states are more pronounced the further away the initial dispersal phenotype is from the fragmentation-adapted short-distance phenotype (e.g. comparing green and red results in figures 2 and 3). This implies that species with historical long-distance dispersal strategies could experience particularly long transient states as they adapt to fragmentation if their dispersal phenotypes are determined by quantitative traits. We modelled four simple initial dispersal kernels but, of course, there are many different dispersal phenotypes in nature. In many species, dispersal kernels are unimodal with a short-distance peak [39]. However, bimodal and even multimodal dispersal kernels are typical of other species, such as wind-dispersed plants with seed dimorphism (e.g. *Crepis sancta* [40]) or plants dispersed by multiple seed-dispersing animals (e.g. [41,42]). Other species, such as many orchids, have a strong tendency for long-distance dispersal, which at least within a substantial range, has an approximately uniform dispersal kernel (e.g. [43]).

There are at least two different mechanisms that can generate long-term transient states which depend on pre-fragmentation dispersal phenotypes and fragmentation rates: (i) evolutionary time needed to cover the phenotypic space distance between initial phenotype and the fragmentation-adapted phenotype and (ii) the dynamics of fragmentation generating stages in which evolutionary transition between particular phenotypes is difficult; for example, transition from long-distance dispersal phenotypes to short-distance dispersal phenotypes requires an intermediate dispersal phenotype in our continuous-shifting model (at least for some part of the dispersal kernel), but intermediate dispersal phenotypes may be particularly unfit in patchy habitats with typical inter-patch distances (which change as fragmentation progresses). These two mechanisms are difficult to tease apart, because both contribute to reduced evolutionary responses in a similar manner in our simulations.

The model parameters we used were not necessarily designed to reflect any real-world system, but we can consider the results from the perspectives of two hypothetical cases: a short-lived plant with life expectancy of 1 year and a long-lived tree with life expectancy of 100 years. Under our parametrization of the model, for the short-lived plant, rapid fragmentation occurs over 9 years, and slow fragmentation occurs over 900 years. For the tree, rapid fragmentation occurs over 900 years and slow fragmentation over 90 000 years. Therefore, in our example, we can estimate that for anthropogenic fragmentation processes with timescales of a few decades to a couple of hundred years, adaptive evolution of dispersal for short-lived plants is relatively unconstrained and will not be affected by the pre-fragmentation dispersal strategies of the population. In fact, short-lived plants have been shown to evolve reduced dispersal in response to fragmentation or isolation within a few generations [44,45]. On the other hand, we demonstrate that the evolution of dispersal in long-living species, such as trees, is limited under rapid and intermediate fragmentation, and therefore for these organisms the ability to adequately adapt to anthropogenic fragmentation would strongly depend on the initial pre-fragmentation dispersal strategy of the species, with historically long-range dispersing species most likely faring the worst. For fragmentation processes occurring over a few decades or years, even the short-lived plants' dispersal strategies may be subject to the evolutionary constraints we describe here. Of course, in order to estimate more realistically the timescale that generates limits to adaptation for any specific system, explicit parametrization of the landscape, demography, fragmentation, and mutation models would be required.

Our continuous-shifting model of the underlying genetics of dispersal traits is simplistic and implicit, with haploid individuals and no interaction between genotypes and environments. However, it captures an important aspect of dispersal evolution. It is important to consider this aspect for dispersal phenotypes that are likely determined by traits such as mass or length, which are most often polygenic. Specifically, for traits in which phenotype space is explored continuously, the evolution of novel distance classes that require trait values significantly different from those which exist in the population would require that the intermediate trait values could be sustained in the population for some time during fragmentation. With rapid fragmentation and polygenic-trait dispersal, this condition is not always met, as demonstrated by the long transient states in figure 2*c*,*f* compared to their absence in figure 3*c*,*f* and figure 2*a*,*b*,*d,e*.

One possible limitation to our model is that the effective number of mutations may depend on the shape of the dispersal kernel, because kernels with stretches of zeros could have ‘synonymous’ mutations (a mutation at a distance class with *d_i_* = 0 and adjacent to distance class $d_{i^{\prime }}=0$ would not affect the dispersal kernel). Therefore, the mutational input is lower with no-dispersal or long-distance dispersal compared to a mutational input in a uniform dispersal kernel. This behaviour may not necessarily reflect the mutational inputs of real dispersal kernels (i.e. an organism with seeds dispersing to many distance classes may not necessarily be able to evolutionarily explore nearby phenotypes faster than organisms with seeds dispersing to a single distance class). However, the similarity in behaviours of the initial uniform dispersal and the initial bimodal dispersal simulations (blue and yellow curves in figure 2, respectively), which differ substantially in the stretches of zeros in their kernels, suggests that the bias in mutational inputs does not play a major role in our results.

Anthropogenic fragmentation is a particularly rapid form of habitat fragmentation, with the dynamic nature of the process playing a crucial role in determining the outcomes. Given climate change and the current rate of human-induced fragmentation, outlining possibilities for rapid adaptation in general and for rapid changes in dispersal traits in particular has received increasing attention with the realizations that such adaptations will be crucial in the ability of organisms to persist in a rapidly changing world [5,46,47]. Nevertheless, most models of the evolution of dispersal under fragmentation have, so far, focused on equilibria in fragmented static landscapes [27], with evolution beginning, progressing and terminating in a static landscape (but see [38]). By and large, modelling efforts concerning the evolution of dispersal kernels predict the evolution of shorter distance dispersal with a decrease in the amount and an increase in the spatial autocorrelation of suitable habitat [25]. This general notion is common to analytical models, as well as to simulation models that use semi-mechanistic [46,48], parametric [17] or distribution-free [20,22] depictions of the evolving dispersal kernel. Our model generates qualitatively similar results in terms of eventual evolutionary response to fragmentation (figure 3; electronic supplementary material, figures S10–S15); the main contribution of our modelling effort is in demonstrating that such adaptive solutions may not come easy or fast in scenarios that are likely to be common in the Anthropocene.

There are still significant gaps in our understanding, however, of the mechanisms and trajectories by which dispersal strategies may evolve, the dependence of dispersal evolution on pre-existing phenotypes, the extent to which they rely on genetic or non-genetic mechanisms [47], and the extent to which they are adaptive. Some of these gaps can be addressed by investigating how dispersal strategies evolve under changing spatial contexts. One study, for example, investigated the case where dispersal strategies that evolve during an early phase of population range expansion become less adaptive at later phases when the spatial context changes, leaving populations entrapped in the range expansion front [37]. Lack of congruency between dispersal strategies, as predicted by current spatial context, and actual adaptation for dispersal have also been empirically demonstrated in range-margin populations [49]. In our model of dynamic fragmentation, the spatial context continuously changes *in situ*, thus generating dynamic selective pressures on dispersal phenotypes. In both population range expansion and dynamic fragmentation, investigations that are based on the assumption of steady states may be missing crucial evolutionary limitations and important long-term transient states. In order to understand the consequences of fragmentation on species, there is a need to consider not only the current ecological circumstances of fragmented populations, but also the pre-fragmentation and evolutionary details affecting dispersal strategies, as well as the historical characteristics of the process that has led to the fragmented landscape.

## Data Availability

The code for all simulations in this paper can be found at github.com/GiliG/Dispersal_Kernel_Evolution.
